# A systematic review of group therapy programs for smoking cessation in Asian countries

**DOI:** 10.18332/tid/140089

**Published:** 2021-08-04

**Authors:** Rashidi Mohamed, Christopher Bullen, Farizah Mohd Hairi, Amer Siddiq Amer Nordin

**Affiliations:** 1 Department of Family Medicine, Faculty of Medicine, National University of Malaysia, Bangi, Malaysia; 2 National Institute for Health Innovation, School of Population Health, University of Auckland, Auckland, New Zealand; 3 Department of Social and Preventive Medicine, Faculty of Medicine, Universiti Malaya, Kuala Lumpur, Malaysia; 4 Department of Psychological Medicine, Faculty of Medicine, Universiti Malaya, Kuala Lumpur, Malaysia; 5 University of Malaya Centre for Addiction Science Studies, Universiti Malaya, Kuala Lumpur, Malaysia

**Keywords:** smoking, group therapy, nicotine, tobacco, cessation

## Abstract

**INTRODUCTION:**

Tobacco causes more than 8 million deaths each year. Behavioral interventions such as group therapy, which provides counselling for smoking cessation, can be delivered in group form and smokers who receive cessation counselling are more likely to quit smoking compared to no assistance. We review the evidence of group-based counselling for smoking cessation for smokers in Asian countries.

**METHODS:**

The review aims to determine the availability of group-based therapy for smoking cessation in Asian countries. The outcome measured was abstinence from smoking following group therapy. Electronic database searches in PubMed, OVID Medline, SCOPUS, Google Scholar, and PsycINFO, using keywords such as: ‘smoking’, ‘cigarette’, ‘tobacco’, ‘nicotine’, ‘group therapy’ and ‘cessation’ (smok*, *cigarette*, tobacco, nicotine, group therap*, cessation) were used. The results were reported following PRISMA and PROSPERO guidelines. Review Manager was used for data analysis.

**RESULTS:**

A total of 21251 records were retrieved for screening the abstracts. In all, 300 articles for review were identified and assessed for eligibility. Nine articles, including Cochrane reviews, randomized control trials, cohort, observational and cross-sectional studies, were included in the final review. There were three observational qualitative studies, two prospective cohort studies, two crosssectional studies, one non-randomized quasi-experimental study and a single cluster-randomized, controlled trial. Group therapy was found to significantly increase the abstinence rate. Group therapy provided at the workplace, smoking cessation services, availability of pharmacotherapy, and socioeconomic status, appear to be key factors determining success.

**CONCLUSIONS:**

Evidence of the use of group therapy for smoking cessation in Asian countries is still lacking despite publications in the Western population showed that group therapy was effective. Further research on group-based interventions for smoking cessation in Asian countries is required and direct one-to-one comparisons between group therapy and individual therapy for smokers who want to quit smoking, are needed.

## INTRODUCTION

Tobacco causes more than 8 million deaths each year worldwide from long-term first hand and secondhand effects of cigarette smoking^1^. Smoking is the act of inhaling and exhaling the fumes of burning plant material. A variety of plant materials are smoked but the act is most commonly associated with tobacco smoked in cigarettes^2^. It is reported that 80% of the total population of smokers worldwide are in low- and middle-income countries^3^. Despite this, smokers in poor countries had no less interest in quitting smoking^4^. Smoking cessation treatment is a vital element in the MPOWER (Monitor tobacco use; Protect people from tobacco smoke; Offer help to quit tobacco use; Warn about dangers of tobacco; Enforce bans on tobacco advertising, promotion and sponsorship; Raise taxes on tobacco) package of tobacco control measures recommended by the World Health Organization (WHO). Most tobacco users want to quit, but only a handful receive support and help to overcome their dependence and the healthcare systems are responsible for treating tobacco dependence. Programs provided by the healthcare system must include tobacco cessation advice, access to medicine, and quitline^5^.

Stopping smoking leads to immediate and long-term benefits such as reduction of risk of stroke among high-risk patients^6^ and premature cardiac deaths among patients^7,8^. The global prevalence of current male smokers is 25% with half of the smokers from Asian countries (China, India, Indonesia). The economic cost of smoking is at a staggering US$ 2 trillion, as most of the cost involves loss of productivity due to smoking-related disease^9^. This amount has not included other collateral damages such as secondhand smoking, agricultural loss of biodiversity, soil erosion, and fire hazards^10^. ASEAN (Association of Southeast Asian Nations) countries have approximately 122.4 million smokers, which is equivalent to 10% of total smokers worldwide^11^. Indonesia has the highest number of smokers in Asian countries^11^. Asian countries are the major contributors of the total number of smokers worldwide. The number of male smokers is much higher than female smokers. In 2019, according to a study by Yang et al.^5,9,12^, the global prevalence of current smoking in men was 25%, and nearly half of the smokers were from China, India, and Indonesia. Among the Asian countries, Indonesia has the highest prevalence of male smokers (76%) followed by Laos (57%), China (48%), Vietnam (47%), Cambodia (44%), Malaysia (43%), Philippines (43%), Pakistan (42%), Thailand (41%), Bangladesh (40%), Nepal (37%), Japan (34%), Myanmar (32%), Singapore (28%), Sri Lanka (28%), South Korea (22%), and India (20%).

A total of about 1.3 billion cigarettes are smoked every day in ASEAN countries. High-income Asian countries like Japan and South Korea have a similar smoking prevalence compared to other developed countries such as Germany. The overall prevalence of smoking in Japan is 19.3%^13^, with predominantly male smokers (26.6%) while 9.3% are female smokers^11^. The overall prevalence of smokers in South Korea with predominantly male smokers did not differ dramatically compared to Japan (19.9% vs 19.3%).

In accordance to the World Health Organization Framework Convention on Tobacco Control (WHO FCTC) Article 14, governments should make smoking cessation easily accessible for would-be quitters. Unfortunately, only a quarter of the 181 WHO FCTC signatories have designated budgets for smoking cessation^14^. Tobacco control interventions have had a positive outcome in high-income Asian countries such as Japan, South Korea and Singapore, but the results have not been replicated in low- and middle-income countries such as China and India^15^. Smoking cessation services in Asian countries vary widely, from almost none, to quit advice at healthcare facilities, to brief intervention, and counselling with pharmacotherapy. In some countries, private pharmacies provide advice on how to quit smoking and in others, telephone quitlines are available^16^. Health workers who have undergone training for smoking cessation are more likely to provide smoking cessation counselling for their patients^17^. People who receive counselling are more likely to quit smoking compared to minimal intervention^18^. Pharmacotherapy, such as nicotine replacement therapy and varenicline, for smoking cessation helps smokers to overcome withdrawal symptoms during the smoking abstinence period, and is of proven effectiveness^19^. It has been estimated that simply providing nicotine replacement therapy (NRT) with the effectiveness of even 1% above baseline in low- and middle-income countries could save nearly 3 million lives over the next century^20^. However, uptake of NRT is low as it is too expensive for many smokers in poor Asian countries, and even when subsidized the uptake of NRT is low^19^. Asian cultures are typically collective and family centered. Hence, group-based social support techniques such as family therapy or ‘buddy’ systems may be of greater interest to smokers than individual treatment^21^. Group-based interventions offer patients the opportunity for social learning, for example sharing knowledge and skills about behavioral techniques for smoking cessation, generate emotional experiences and provide mutual support^22^. Evidence has also shown that group therapy for smoking cessation had demonstrated preliminary efficacy and feasibility of group-based smoking cessation treatment with pharmacotherapy in a special population^23^.

Furthermore, group-based approaches may be a more efficient way of reaching and supporting the many millions of Asian smokers who need support to quit than current individually targeted approaches. In some settings, group treatment has been shown to be more effective than no intervention or minimal intervention and about as effective as an intensive individual intervention^24^ but more affordable^25^. In this systematic review, our objective was to examine the evidence on the availability of group therapy as a behavioral intervention for smoking cessation for smokers who want to quit smoking in Asian countries and the documented abstinence after a quit attempt.

## METHODS

We conceptualized the review by setting various objectives related to the subject of behavioral support, particularly group therapy, in Asian countries. The objectives were to determine the abstinence rate among patients in group therapy as a behavioral intervention for smoking cessation and to compare the effectiveness of group-based therapy for smoking cessation available for smokers to quit smoking in Asian countries. Abstinence is defined as no use of combustible cigarettes, without considering the use of other tobacco or alternative products^26^ and not smoking for 3 to 6 months from the quit date. The study population in this systematic review are smokers who have joined a group therapy as a behavioral intervention for smoking cessation conducted in Asian countries with abstinence from smoking as the outcome of interest.

The behavioral intervention has been frequently used to help smokers to quit smoking but the effectiveness and content of the intervention vary substantially. To identify the eligibility of the studies included in the systematic review, we searched and reviewed the articles with the keywords: ‘smoking’, ‘cigarette’, ‘tobacco’, ‘nicotine’, ‘group therapy’, and ‘cessation’ (smok*, *cigarette*, tobacco, nicotine, group therap*, cessation). We selected studies to be included in this systematic review based on inclusion and exclusion criteria. The key criteria used during the assessment of the type of study selected in this systematic review were: recruitment, treatment allocation, randomization, response rate, outcome measurement, levels of missing data, and how missing data were addressed. In our systematic review, studies with participants who were cigarette smokers aged ≥18 years, articles from 1 January 2004 to 6 July 2020 published in English only were included. The databases searched were PubMed, OVID Medline, SCOPUS, Google Scholar and PsycINFO. We also looked if the reported smoking cessation abstinence measurement of cessation used biochemical validation at the reported time point. Studies with incomplete data or estimates were excluded from the analysis. Studies with low grade of evidence were included only after discussion among the researchers. A third reviewer was consulted when an agreement could not be reached between the two researchers. Various study designs including systematic reviews, qualitative studies, cross-sectional observational studies, longitudinal observational studies, prospective randomized controlled trials, and other experimental studies, were evaluated for inclusion in this systematic review.

A variety of behavior therapies ranging in complexity from simple advice offered by a physician or other healthcare provider or a much more extensive therapy have been shown to be efficacious for tobacco smoking cessation. The success rate for abstinence from smoking increases when behavioral therapy is combined with pharmacotherapy. A behavioral intervention involves discussion, encouragement, advice and other modalities to help to achieve behavioral change^19^.

Group therapy is defined as the process of giving and receiving assistance, from individuals with similar conditions or circumstances, to achieve recovery in a group form. The group of people in group therapy voluntarily gather to receive support and provide support by sharing knowledge, experiences, coping strategies, and offering understanding towards smoking cessation intervention. The most common behavioral intervention for smoking cessation was individual therapy. Individual therapy is a face-to-face session with a trained therapist that focuses on behavioral change, which also incorporates motivational interviewing^27^. The individual intervention involves self-exploration and identifying ambivalence so that resolutions can be determined for effective behavioral change^28^.

Strategies for helping smokers to quit include behavioral counselling to enhance motivation and to support attempts to quit and pharmacological intervention to reduce nicotine reinforcement and the withdrawal symptoms of cessation of tobacco use^29^. We have included all the studies that fulfilled the inclusion and exclusion criteria. We evaluated the studies included in the systematic review by looking at the nature of behavioral support provided such as motivation to quit smoking, mode of delivery of the behavioral support, behavioral intervention service provider, and presence and type of pharmacotherapy provided.

The study domain was behavioral intervention (group therapy) for the treatment of nicotine addiction secondary to cigarette smoking. The outcome measure was abstinence from smoking following different types of behavioral interventions with or without pharmacotherapy. Abstinence was defined as not smoking for 3 to 6 months from the quit date. The results of the systematic review are reported following both the Preferred Reporting Items for Systematic Reviews and Meta-Analysis (PRISMA)^30^ and the International Prospective Register of Systematic Review (PROSPERO)^31^ guidelines. Articles were excluded if other forms of tobacco were involved, such as chewing tobacco, electronic devices such as e-cigarettes, and other drug use such as cannabis.

Various study designs selected including systematic reviews, qualitative studies, cross-sectional observational studies, longitudinal observational studies, prospective randomized controlled trials, and other experimental studies, and the intervention effect (group therapy for smoking cessation) was measured by before and after treatment estimates that provided important information on the outcome. Review Manager (RevMan) software (version 5.4, Copenhagen: Nordic Cochrane Centre, Cochrane Collaboration) was used for data analysis^32^. We used the random-effects model. Heterogeneity between studies was assessed using the I^2^ test. An I^2^ value of 0% indicates no observed heterogeneity, and larger values show increasing heterogeneity (75% or greater considered substantial heterogeneity).

## RESULTS

**Figure 1:**
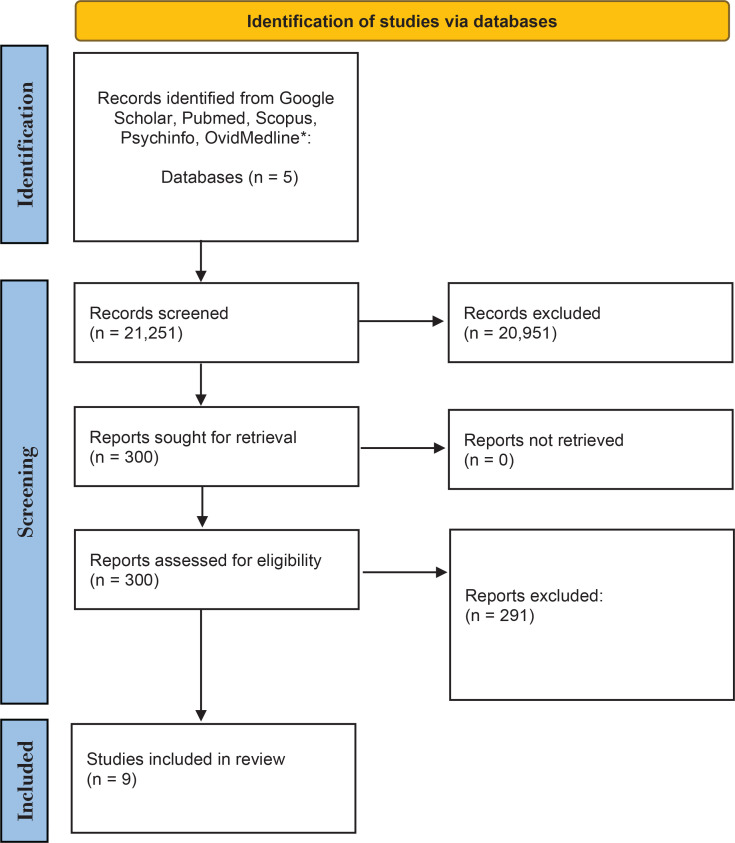
Flow chart of search strategy results of electronic database search which include Google Scholar, PubMed, Scopus, PsycINFO, Ovid Medline from years 2004–2020

After the screening we identified a total of 300 articles for review (Figure 1). They were assessed for eligibility by checking against the inclusion and exclusion criteria, leaving 9 articles for the final systematic review. The selected journals were studies conducted in Asian countries, published in English, with full-text article available. All the selected studies for this systematic review fulfilled the inclusion criteria. Table 1 shows that the nine studies in the systematic review were in middle- and high-income Asian countries (Malaysia, India, China, Taiwan, Iran, Mongolia, Pakistan, Japan, and South Korea)^6,14,24,33-38^. Five studies were conducted in healthcare centers with smoking cessation clinics, three at universities, and one at a factory (a workplace intervention). The original authors were contacted to obtain further information for studies where details related to the systematic review were missing. There were three observational qualitative studies, two prospective cohort studies, two cross-sectional studies, one non-randomized quasi-experimental study, and a single cluster-randomized, controlled trial. The type of intervention (pharmacotherapy + behavioral therapy, pharmacotherapy only, behavioral therapy, or no intervention) varied between the selected studies. Six studies provided pharmacotherapy and behavioral intervention and three provided behavioral support. Four studies described using pharmacotherapy (nicotine replacement therapy) and behavioral therapy, and only one study described the use of bupropion. Behavioral therapy only, was provided in three studies. The studies included in the systematic review included smokers who smoked at least one cigarette per day with a mean of 10–22.1 cigarettes smoked per day, and low to high level of nicotine dependence. (Figure 1).

**Table 1:** Summary of the data extracted from articles identified in systematic review comparing group and other smoking cessation interventions services among Asian countries

	*First author, year of publication and country where study was conducted*	*Role of group therapy*	*Types of study*	*Treatment type/comparison*	*Details of methodology*	*Number of participants*	*Results*	*Conclusion*	*Quality of evidence (Grade)*
**1**	**Maarof 2016 Malaysia**	Group therapy as a behavioral intervention for smoking cessation	Qualitative Observational study	Group therapy for two separate groups	Evaluating a developed module for smoking cessation in a single focus group discussion. Suitability of the module was assessed by using a questionnaire with a Likert-scale and behavioral issues that were identified as themes were included in developed module.	8 (4 each group)	Seven major themes: reasons for regular smoking, reasons for quitting, comprehending smoking characteristics, quit attempt experiences, support and encouragement, learning new skills and behavior, and preparing for lapse/relapse or difficult situations.	Findings indicate that components developed were important and could be applied in delivering group behavioral therapy.	Low
**2**	**Baigalmaa 2006 Mongolia**	Group therapy as a behavioral intervention for smoking cessation Behavioral therapy only	Prospective cohort study	Group therapy and face to face with follow-up telephone consultation	Each group consisted of 12-16 participants. Training included information on tobacco or health, needs to quit, ways to overcome smoking behavior, problem solving and individual plans for behavioral modification, adjusting to become non-smoker and setting a quit date. Participants were followed up by telephone at 1, 3, 6, and 12 months. Cessation program and follow-up period, educational materials and consultations were provided for the participants.	517	The cessation rate of 2 years during the follow-up period gradually decreased from 70.6% at the first month to 65% at the 12th month. Behavior modification among heavy smokers after 12 months was 47%.	Group counselling for smoking cessation is effective for smokers with an intention to quit smoking. Group programs were more effective for helping people to stop smoking than being given self-help materials without face-to-face instruction and group support.	Moderate
**3**	**Huang 2005 Taiwan**	Short-term group support for smoking cessation Pharmacotherapy and behavioral therapy	Qualitative observational study	Group therapy with follow-up telephone consultation	Develop and evaluation of outcomes of a smoking cessation program with combination of physiological and psychological treatment in a group. Three-month program with three monthly group sessions, pharmacotherapy (free nicotine patches) and telephone counselling.	10	Significant reduction in %COHB level and number of cigarettes smoked at data-point, and in the number of cigarettes smoked at one month from the pretest, at the 3 months test and at the 9 months follow-up. At the 9 months follow-up, 50% abstinent rate, and 30% had decreased cigarette consumption by at least 49% of their pretest levels. 80% has changed their smoking behavior.	There is a need for an integrated group support for smoking cessation at a larger scale.	Moderate
**4**	**Avaisu 2011 Malaysia**	Face-to-face behavioral intervention for smoking cessation Pharmacotherapy and behavioral intervention	Non randomized quasi-experimental study	Effectiveness of smoking cessation intervention among tuberculosis patients receiving SCIDOTS vs standard care for TB treatment (DOTS)	Comparison between conventional TB DOTS plus smoking cessation intervention (integrated intervention or SCIDOTS group) or conventional TB DOTS alone (comparison or DOTS group)	120	120 eligible participants who were current smokers at the time of TB diagnosis were assigned to either of two treatment groups: 7-day point prevalence abstinence and continuous abstinence was observed over time in the intervention group. At the end of 6 months, patients who received the integrated intervention had significantly higher rate of success in quitting smoking when compared with those who received the conventional TB treatment alone.	Face-to-face smoking cessation intervention provided with DOTS for tuberculosis patients show that patients who received the intervention had significantly higher success rate in quitting smoking compared to standard care.	Moderate
**5**	**Siddiqi 2013 Pakistan**	Group therapy in the form of focus group as a behavioral support Pharmacotherapy and behavioral therapy	Cluster randomized, controlled trial	Patient randomized into 3 groups [behavioral support sessions (BSS), BSS plus 7 weeks of bupropion therapy or usual care] and primary and secondary endpoint was measured. (Primary end point was continuous abstinence at 6 months after the quit date. Secondary end points were point abstinence at 1 and 6 months)	Suspected tuberculosis patients who come to the health centers were screened for smoking and were randomized into 3 groups (BSS plus, BSS only and usual care). This is a balanced, pragmatic, cluster randomized trial with 3 groups. Patients in one group received 2 brief BSS (BSS group), patients in the second group received 2 brief BSS plus 7 weeks of bupropion therapy (BSS group), and patients in the control group received usual care. All patients receive self-help printed materials.	1955	Behavioral support, alone or in combination with bupropion, was effective in achieving continuous smoking abstinence at 6 months compared with usual care [RR for BSS plus, 8.2 (95% CI: 3.7–18.2); RR for BSS, 7.4 (95% CI: 3.4–16.4)] Relative risks (RRs) for abstinence compared with usual care [RR for BSS plus 8.2 (95% CI: 3.7–18.2); RR for BSS, 7.4 (95% CI: 3.4–16.4)]. For continuous abstinence, BSS plus group achieved higher 45.4% (95% CI: 41.4–49.4) compared to BSS (41.0%).	The estimated cost of behavioral support ($2.50 per participant) was approximately one tenth that of behavioral support plus bupropion ($20.90 per participant). Low- and middle-income countries, where access to and afford- ability of medicine is constrained, might favor an inexpensive non-pharmacological intervention that can be delivered by existing staff. However, BSS+ vs BSS alone in a non inferiority analysis cannot be confirmed. BSS can be a Best Buy to reduce smoking prevalence and NCDs in low- and middle- income countries.	Moderate
**6**	**Sharifi 2012 Iran**	Group therapy as part of harm reduction intervention for smokers in Iran Pharmacotherapy and behavioral therapy	Prospective cohort study	Patients were assigned to groups (5 to 15 members in each group) in conjunction with the use of nicotine gum and followed up at 2, 4, 6, 8 and 10 weeks following study initiation	The study was conducted for 12 months among patients who were unable to quit. Patients were informed regarding smoking reduction and abstinence. Primary outcome was to evaluate abstinence and smoking reduction at the third and sixth months of follow-up: the number of smoked cigarettes, level of expired carbon monoxide (CO), and numbers of nicotine gum used.	132	64.4% of the study participants reduced the number of daily smoked cigarettes by at least 50% and 12.9% quit smoking at 6 months.	Smoking reduction and abstinence can be achieved by prolonged counselling and NRT. Smoking reduction is a useful method for smokers who are unable to stop smoking immediately.	Low
**7**	**Lee 2017 South Korea**	Group therapy for positive psychotherapy and motivational interviewing as an intervention for smoking cessation Behavioral therapy only	Observational Qualitative study	Positive group psychotherapy and motivational interviewing were attended by 36 smokers for 1 hour once a week, for 6 hours (with a total of 6 sessions in 6 weeks) followed by follow-up at 3 months and at 6 months. An interview was conducted after the intervention was completed	36 study subjects were recruited. The importance of smoking cessation was higher among the group of participants who managed to successfully quit smoking at 3 months (n=10) and participants who did not have a successful quit attempt at 3 months (n=26)	36	The confidence to stop smoking was rated higher by the success (p<0.01). The reason for a successful quit attempt was for their loved ones (60%) and health (50%). The unsuccessful group wanted to stop smoking to save money (45.5%). The unsuccessful group had more than 1 cross addiction compared to successful group. They also had less participants with 10 best personal merits than the successful group.	The importance of motivation and confidence in smoking cessation were predictors for successful cessation. Motivational interviewing increased motivations, whereas positive group psychotherapy increased positive thoughts and confidence, which are essential for a successful quit attempt.	Low
**8**	**Hotta 2007 Japan**	Effectiveness of group therapy in university and workplace environment to assist smokers to quit smoking Pharmacotherapy and behavioral therapy	Cross-sectional study	Testing the efficacy of group therapy for the participants who want to quit smoking in Okayama University, Japan. This program consisted of behavioral support, nicotine patches and online support. Smoking status was assessed by direct interviews. A total of 7 visits of counselling and medication were provided.	A total of 102 employees were enrolled in the cessation program, which corresponding to 20.4% of the total smokers in the university. Majority were male, aged ≥20 years, smoked 23 cig/day, had moderate dependence to nicotine and had median of 16 ppm in CO Smokerlyzer reading.	102	Out of the 102 participants, 1 refused to participate in the study after registering, 7 did not turn up for follow-up after 1 year. 53% of the remaining 94 participants had obtained abstinence. In the intention-to-treat group, where participants who were lost from follow-up are considered as smoking, the cessation rate was calculated as 50% (50/101).	Type of position was a significant factor affecting the 1-year cessation rates with 78%, 55% and 6% in the academic, administrative and technical staff. It was reported that type of position at workplace and sending email within the first week of cessation attempt was a significant factor affecting the 1-year cessation rates.	Moderate
**9**	**Pimple 2014 India**	Behavioral intervention (individual + group therapy) for smoking cessation at workplace Behavioral therapy only	Cross-sectional study	There were 3 sessions provided for a duration of 6 months (0, 3, 6). Workers are divided into groups with a limit of 15–45 persons per group. The sessions were conducted under the principles of group therapy. The sessions were: supportive psychotherapy, cognitive behavior therapy, and psychodrama. There was no pharmacotherapy provided. The attrition rate of 78.6% (176) was achieved.	Majority of the participants were in precontemplation phase. After 3 months, 59 users remained at the contemplation phase, and an increase from 21 to 52 for preparation, from 21 to 95 for action and maintenance, and 6 relapsed, compared to post-intervention assessment. At 6 months, 57 participants remained at the contemplation phase, while the number decreased to 45 for preparation, from 95 to 38 for action and maintenance, and 36 relapsed, compared to post- intervention II assessment. Factors such as sociodemographic characteristics and smoking status did not influence the intention to quit, however, presence of pre-cancerous oral lesions during screening has been found to be a factor for quit attempt. Like age, gender, education, income, marital status, religion, alcohol use, personal medical history, Fagerström score, previous quit attempts, forms of tobacco use, withdrawal symptoms experienced and family members tobacco history had no bearing on their intent and decision to quit. In contrast, presence of clinical oral pre-cancer lesions found to be associated with quitting.	224	Majority of the workers, 95 (42.4%), who successfully quit in the initial stages post second intervention program were not able to follow the linear path to maintenance. The current study witnessed around 6 (2.7%) followed by 36 (16.1%) workers relapsing at the end of second and third (last) intervention sessions. Extended cessation therapies with relapse prevention strategies may help combat the problem.	Behavioral intervention at workplace is a cost- effective tool to help smokers to quit smoking. increase the likelihood of quitting. Employers can maintain a smoke-free workplace by promoting tobacco control measures for overall health benefits of the employees	Low

**Figure 2:**
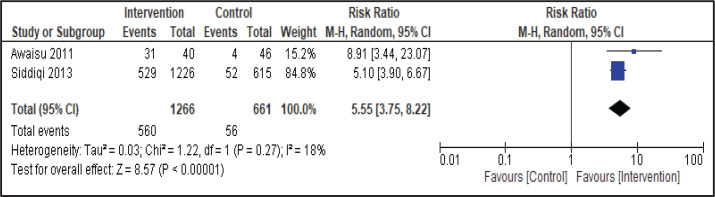
Forest plot of intervention study by Awaisu 2011 and Siddiqi 2013 on behavioral therapy with pharmacotherapy versus usual care in tuberculosis patients: effect on continuous abstinence of smoking at 6 months

Two studies involving group therapy as behavioral intervention in the smoking cessation treatment compared outcomes with usual care practice. In a pooled analysis of these studies using the random-effects model, the intervention group significantly increased the abstinence rate (Figure 2). A total of 560 of 1266 (44.2%) patients who received intervention had quit smoking at 6 months compared with 56 of 661 (8.5%) patients who received usual care (RR=5.55; 95% CI: 3.75–8.22, p<0.001). In the study involving smokers who had tuberculosis and had undergone group therapy as smoking cessation behavioral support, the number of participants included in the final analysis was equal and comparable between the two groups. However, a heterogeneity analysis was conducted and there was no significant heterogeneity: I^2^=18% (p=0.27) (Figure 2).

## DISCUSSION

Our review identified an important finding in the treatment for cigarette smoking for smokers in Asian countries. The availability of group therapy as an alternative to individual therapy (standard care) provides a treatment option for smokers to choose when a smoker decides to quit smoking. Furthermore, evidence has shown that group therapy provides better outcomes compared to minimal intervention or no intervention. Despite the evidence published in the western population, we found only a handful of articles relevant to our question of interest, which was the availability of group therapy for smoking cessation in Asian countries (Table 1). The studies looked at the use of group therapy in various circumstances such as group therapy + pharmacotherapy, counselling in the form of group therapy, and the treatment outcome.

Most of the studies selected in this systematic review were conducted in middle-income (Malaysia, Pakistan, India, Mongolia, Iran) and high-income countries (Japan, South Korea). In general, less wealthy countries have fewer resources to invest in smoking cessation than higher income countries. Despite this, smokers in poorer countries had no less interest in quitting smoking. Although smokers in middle-income countries were reported to have lower use of quit smoking medication and healthcare services, it does not translate to less interest in quitting. In Malaysia, smokers are keen to respond to healthcare queries on smoking behaviours^7^. This is important, because 80% of the world’s smokers are from low- and middle-income countries and it is estimated that 7 million deaths attributable to smoking will occur by 2030^15^.

Huang et al.^34^ reported high abstinence, reduced number of cigarettes smoked and change in smoking behavior in group intervention with pharmacotherapy. Meanwhile, Siddiqi et al.^36^ found that group therapy alone or in combination with pharmacotherapy (e.g. bupropion) was effective. Sharifi et al.^37^ reported that counselling and pharmacotherapy can achieve smoking abstinence and reduction of the number of cigarettes smoked per day, as the smoking reduction was found to be a useful method for smokers who are unable to stop smoking immediately. Despite their potential among Asian smokers, group-based interventions for smoking cessation are under-researched. In other settings, studies have shown group-based treatment interventions to be effective. Group treatment that included medication such as varenicline, NRT, and bupropion, or bupropion + NRT, decreased the number of cigarettes smoked per day in a single group behavioral support^39^. Our results align with those of reviews in western nations, such as studies by Prochaska et al.^40^ and Schlam et al.^41^, in which behavioral support with pharmacotherapy increased cessation rates and improved long-term abstinence, but most smokers eventually relapsed.

Combining behavioral interventions such as counselling and pharmacotherapy for smoking cessation helps smokers in their quit attempt and the outcome is better than counselling alone, even if the counselling is provided by healthcare professionals. Behavioral support with pharmacotherapy increased cessation rates and improved long-term abstinence, but most smokers eventually relapsed^42^. There should be monitoring and supervision by healthcare professionals and the management should be collaborative work between doctors and nurses. Quit smoking initiatives at universities and workplaces have proven to be an effective setting for early quit attempts^43^ and should be explored in Asian nations. Employers should not only promote smoke-free workplaces and provide incentives to motivate smokers to quit smoking, but also introduce group counselling to help employees quit cigarette smoking.

## CONCLUSIONS

Despite its potential and some evidence of benefit, research on group-based interventions for smoking cessation in Asian countries is lacking. Direct one-to-one comparisons between group therapy and individual therapy in behavioral support for smokers who want to quit smoking are needed. Innovative setting-based studies are also needed, such as those exploring the potential of workplaces and other group settings. Such evidence, based on the efficacy, affordability and feasibility of group therapy for smoking cessation among Asian smokers, would then support country-specific national guidelines to optimize country-specific and cost-effective smoking cessation initiatives. The practical implication identified in this systematic review on group therapy is that it can increase the likelihood of quitting cigarette smoking when the person is motivated in joining the group therapy. Group therapy can become a comprehensive extension of standard care available in most healthcare system. The impact of group therapy can become meaningful depending on the uptake so that the benefit of group therapy such as cost-effectiveness can be observed and documented. For research implications related to this systematic review, a larger number of participants in group therapy for smoking cessation would be desired to identify the impact of specific components in group therapy research and its efficacy.

## Data Availability

The data supporting this study are available from the authors on reasonable request.
